# Functionalities of electrochemical fluoroquinolone sensors and biosensors

**DOI:** 10.1007/s11356-023-30223-2

**Published:** 2023-12-19

**Authors:** Collen Nepfumbada, Nomcebo H. Mthombeni, Rudzani Sigwadi, Rachel F. Ajayi, Usisipho Feleni, Bhekie B. Mamba

**Affiliations:** 1 Institute for Nanotechnology and Water Sustainability (iNanoWS), College of Science, Engineering and Technology (CSET), University of South Africa (UNISA), Florida Campus, Johannesburg, 1709 South Africa; 2 Department of Chemical Engineering, Faculty of the Built Environment, Durban University of Technology, Steve Biko Campus, Durban, 4001 South Africa; 3 Department of Chemical Engineering, University of South Africa (UNISA), Florida Campus, Johannesburg, 1709 South Africa; 4 SensorLab (University of the Western Cape Sensor Laboratories), 4th Floor Chemical Sciences Building, University of the Western Cape, Robert Sobukwe Road, Bellville, Cape Town, 7535 South Africa

**Keywords:** Fluoroquinolones, Antibiotics, COVID-19, Biosensors, Detection, Nanomaterials

## Abstract

Fluoroquinolones (FQs) are a class of broad-spectrum antimicrobial agents that are used to treat variety of infectious diseases. This class of antibiotics was being used for patients exhibiting early symptoms of a human respiratory disease known as the COVID-19 virus. As a result, this outbreak causes an increase in drug-resistant strains and environmental pollution, both of which pose serious threats to biota and human health. Thus, to ensure public health and prevent antimicrobial resistance, it is crucial to develop effective detection methods for FQs determination in water bodies even at trace levels. Due to their characteristics like specificity, selectivity, sensitivity, and low detection limits, electrochemical biosensors are promising future platforms for quick and on-site monitoring of FQs residues in a variety of samples when compared to conventional detection techniques. Despite their excellent properties, biosensor stability continues to be a problem even today. However, the integration of nanomaterials (NMs) could improve biocompatibility, stability, sensitivity, and speed of response in biosensors. This review concentrated on recent developments and contemporary methods in FQs biosensors. Furthermore, a variety of modification materials on the electrode surface are discussed. We also pay more attention to the practical applications of electrochemical biosensors for FQs detection. In addition, the existing challenges, outlook, and promising future perspectives in this field have been proposed. We hope that this review can serve as a bedrock for future researchers and provide new ideas for the development of electrochemical biosensors for antibiotics detection in the future.

## Introduction

Antibiotics can be described as either natural or synthetic compounds with useful antibacterial activities that are usually employed in human and veterinary medicine to treat various infectious diseases (Khan 2020; Cardoso et al. 2021). Overuse of antibiotic drugs could lead to bacteria resistance, creating challenges to societies and health centers due to increased patient numbers and costly treatment (Yadav et al. 2021). Antibiotics are categorized according to their mechanism of action or chemical structure and are arranged into classes that include quinolones, ß-lactams, sulphonamides, macrolides, and tetracyclines (Hamnca et al. 2017; Ding et al. 2021). Table 1 shows the common antibiotics and their properties. Among these antibiotics, quinolones such as Fluoroquinolones (FQs) have gained significant interest due to their widespread application in households, hospitals, and veterinary for the treatment of infectious diseases (Teglia et al. 2019). Over the past four years, there has been an increase in the use of FQs due to the COVID-19 pandemic as there is no evidence of any specific recommended treatment measures for patients with confirmed COVID-19 (Miranda et al. 2020; Ebrahimi and Akhavan 2022). As a result, FQs are frequently detected in different environmental compartments due to an incomplete metabolism in the target organism and inefficient wastewater treatment (Cuprys et al. 2018; Gou et al. 2021), leading to accumulation of these drugs in human bodies through drinking water, which in turn poses serious detrimental health effects to both humans and the environment (Gaudin 2017; Kraemer et al. 2019; Lan et al. 2017). Hence, to prevent further antibiotic contamination, national governments should limit antibiotic use in livestock and aquaculture (Ters 2022). In this outlook, we are yet to find the report exploiting safe concentration for commonly used antibiotics in water regulated by national governments to ensure safety for living organisms. Thus, there is a need for the development of new reliable approaches for detecting antibiotics and their metabolites in the environment to ensure public health safety.

**Table 1:** Detailed description of different antibiotics and their general characteristics

Type of antibiotics	Functional groups	Target microbes	Mechanism of action	Example	Chemical formula	Side effects	Application	Ref
Tetracycline	Four hydrocarbon rings (cyclins)	Both gram-positive and negative bacteria	Inhibition in protein synthesis through binding with ribosome	Tetracycline Oxytetracycline Doxycycline	C_22_H_24_N_2_O_8_ C_22_H_24_N_2_O_9_ C22H_24_N_2_O_8_	Hepatotoxic	Human, veterinary, human,	(Liu et al. 2018)
Sulphonamides	The Sulfonyl group (O = S = O) is connected to an amine group (− NH2)	Both gram-positive and negative	Inhibition in folic acid synthesis	Sulphanilamide Sulfamethoxazole Sulfadiazine	C_6_H_8_N_2_O_2_S C_11_H_11_N_3_O_2_S C_12_H_14_N_4_O_4_S	Diarrhea, vomiting, and nausea	Veterinary, veterinary, human	(Zhou et al. 2022)
Fluoroquinolones	Bicyclic core structure	Gram-negative bacteria	Inhibition of bacterial DNA Gyrase	Ciprofloxacin Norfloxacin Levofloxacin	C_17_H_18_FN_3_O_3_ C_16_H_18_FN_3_O_3_ C_18_H_20_FN_3_O_4_	Hepatic toxicity, haemolytic anemia	Human, veterinary, human, human,	(Bhatt and Chatterjee 2022)
β-lactams	Lactam ring 3-C and N ring	Both gram-positive and gram-negative	Inhibition of peptidoglycan layer	Penicillin Amoxicillin Cephalosporins	C_16_H_18_N_2_O_4_S C_16_H_19_N_3_O_5_S C_16_H_21_N_3_O_8_S	Rashes, fever	Human, veterinary	(Maciel et al. 2023)
Macrolides	14;15;16 membered macrocyclic lactone ring	Streptococcal/pneumococcal	Inhibition in bacterial protein biosynthesis	Erythromycin Clarithromycin Roxithromycin	C_37_H_67_NO_13_ C_38_H_69_NO_13_ C_41_H_76_N_2_O_15_	Gastrointestinal disturbances	Human, veterinary	(Nguyen et al. 2023)

According to the published literature and national studies, the concentrations of pharmaceutical products in surface and groundwater impacted by wastewater discharges are typically less than 0.1 µg L^−1^ (or 100 ng L^−1^) as shown in Table 2, whereas the concentrations in treated drinking water are usually well below 0.05 µgL^−1^ (Maycock and Watts 2011). There are not many thorough, systematic research on the presence of pharmaceutical compounds in drinking water. Hence, assessing possible dangers to human health from exposure to trace amounts of these compounds in drinking water is difficult due to the lack of data on the topic (Epa 2008). As a result, there is no evidence yet on the standard safety of these antibiotics in water.

**Table 2:** Summary of concentrations levels of FQs in different streams

Fluoroquinolones	Streams	Concentrations	Refs
Ciprofloxacin	Tap water	6.0–679.7 ng L^−1^	(Yiruhan et al. 2010)
Levofloxacin	River	42 ng L^−1^	(Speltini et al. 2015)
Ofloxacin	Wastewater	9 ng L^−1^	(He et al. 2015)
Enrofloxacin	Water sample	0.003 μg L^−1^	(Zhang et al. 2013)
Norfloxacin	Water sample	3.02–23.90 ng mL^−1^	(Madikizela et al. 2022)
Enrofloxacin	River and lake samples	0.22 μg L^−1^	(He et al. 2015)
Ciprofloxacin	Water	127 µg L^−1^	(Ajibola et al. 2021)

Several techniques have been reported for FQs detection in different samples, including high-performance liquid chromatography (HPLC) (Abedalwafa et al. 2019), liquid chromatography–mass spectrometry (LC–MS) (Lim and Ahmed 2016), capillary electrophoresis (CE) (Zhang et al. 2020), and immunoassay (Acaroz et al. 2020). Although these methods are sensitive, they are often costly and time-consuming and usually require specialized/skilled personnel to operate, which in turn limits their potential application. For these reasons, conventional methods are not suitable for routine and rapid analysis of large numbers of samples (Wu et al. 2016; Kharewal et al. 2020). Consequently, new approaches are needed to overcome the limitations of the traditional methods.

Recently, with the rapid increase in nanotechnology, electrochemical methods on specific biometric elements have been extensively used for FQs detection owing to their advantages such as low cost, rapid response, high sensitivity, easy operation, and suitability for on-site monitoring (Jahanbani and Benvidi 2016). Electrochemical biosensors have emerged as an alternative strategy for antibiotics detection. Biosensors are a group of state-of-the-art analytical devices that use a biorecognition material in close contact with a transducer. The recognizing elements such as enzymes, antibodies, and DNA are the most used when developing biosensor (López et al. 2017; Yazdanparast et al. 2019). The binding affinity of a recognition element with a target analyte plays a critical role on biosensor performance. The greatest challenges associated with biosensor development involve the efficient capturing of biorecognition signals and the transformation of these signals into either electrochemical, electrical, and optical signal. However, one of the recent trends to overcome such drawbacks related to biosensor fabrication is through the integration of sensing technology and nanomaterials (NMs) with properties such as high surface-to-volume ratio, good conductivities, shock-bearing abilities, and color tunability (Kumar and Neelam 2016; Lawal 2018). These NMs have a high capacity for charge transfer and influence the biosensor to produce high sensitivity and lower detection limit (LOD). Herein, this review reports the recent advances in electrochemical biosensing systems for FQs detection water sample. To this end, the electrochemical biosensor for FQs detection and their development were briefly introduced. The review also discusses the challenges encountered by the existing electrochemical biosensor and how their performance can be improved further. Therefore, the concepts introduced in this review are expected to motivate new findings toward electrochemical detection of FQs in future.

## Fluoroquinolones (FQs) as environnemental pollutants

**Fig. 1:**
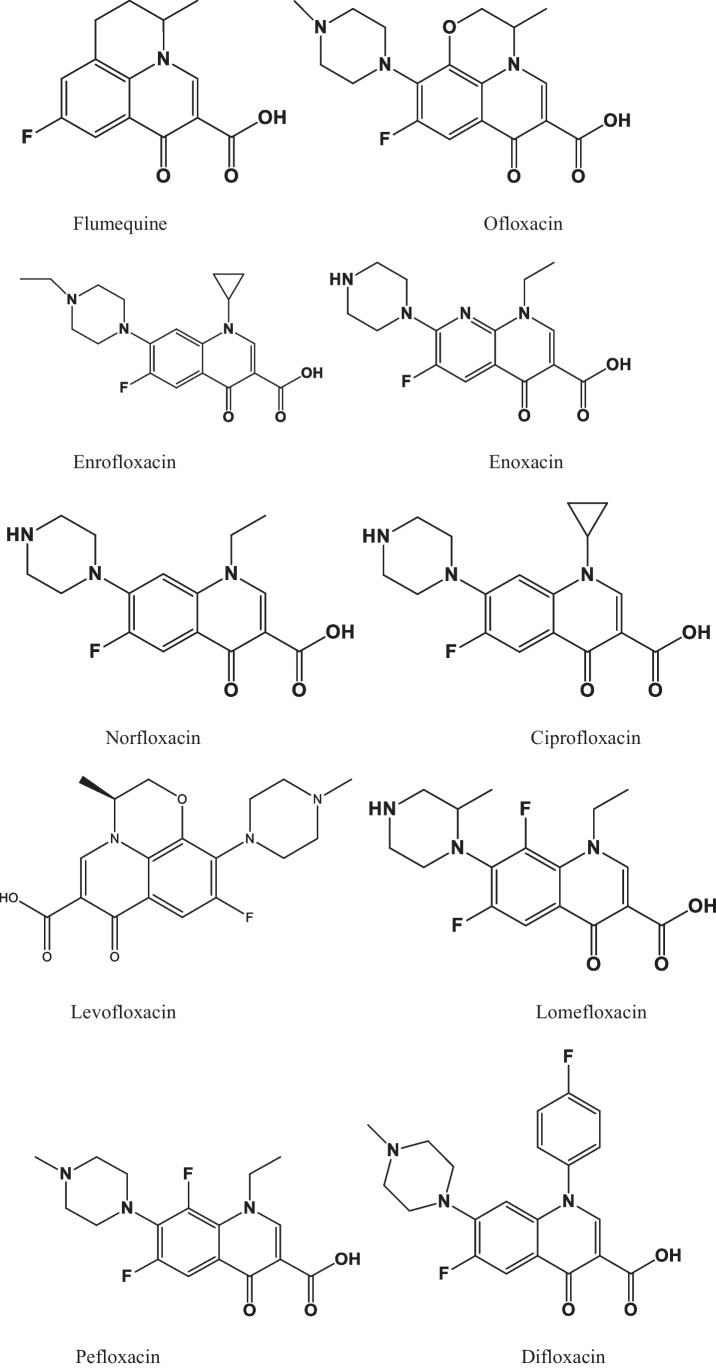

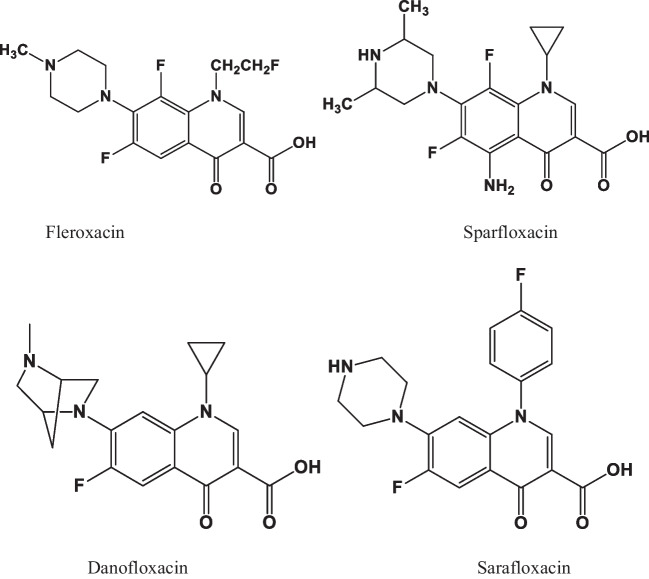
Structures of fluoroquinolone antibiotics

Fluoroquinolones (FQs) are a synthetic group of antimicrobial agents, which are derived from quinolone nalidixic acid by the addition of a fluorine atom at carbon 6 and piperazine at carbon 7 position (Zahra et al. 2021). FQs are considered as broad-spectrum antibiotics due to their bactericidal effect against various pathogenic bacteria. They destroy the bacteria or inhibit their growth by inhibiting their DNA gyrase replication favored by their chemical structure (Rasheed et al. 2023). Since the discovery of using FQs for the treatment of living organism, a number of FQs are being prescribed for their broad-spectrum mode of action toward Gram-negative, Gram-positive bacteria, and mycoplasma (Zhang et al. 2018). However, the extensive use of FQs leads to an accumulation of these compounds in aquatic and terrestrial environments in large quantities, which could probably cause allergic reactions in humans and the emergence of food-borne bacteria (Lu et al. 2021a). Releasing effluents from different manufacturing sectors is one of the most significant pathways for these drugs to enter the aquatic ecosystem (Bhatt and Chatterjee 2022). On the other hand, humans and animals partially metabolize FQs, and about 10–70% of these drugs are excreted and released into the sewage and subsequently enter the wastewater treatment plants (WWTPs) (Sodhi and Singh 2021; Sodhi et al. 2021). FQs and their metabolites are highly toxic and the continuous discharge of these drugs into the water bodies can pose potential risks to aquatic organisms and marine biodiversity even at lower concentrations (Ramesh et al. 2021). In addition, when the antibiotics interact with bacteria in water bodies, new pathogenic species are being formed that are resistant to these compounds (Leibovici et al. 2016; Cristea et al. 2017; Majdinasab et al. 2017). According to World Health Organization (WHO), the increase in antibacterial resistance has become a national and international issue that threatens society’s health by spreading antibiotic-resistant bacterial infections (Flaherty and Cummins 2017). FQs are classified as first-generation, second-generation, third generation, and fourth-generation agents to describe their evolution based on the antibacterial spectrum. However, the most used FQs antibiotics belong to the second and third generation. Thus, the occurrence of FQs in surface water and wastewater has drawn great attention. Figure 1 shows the different types of FQs antibiotics.

## The analytical method used for FQs antibiotics detection

The monitoring and screening of FQs in water is imperative as it is the first stage to dealing with environmental pollution generated by these antibiotics. Several conventional technologies have been employed and reported for the detection of FQs in water bodies. This includes spectrofluorometry, spectrophotometry, photochemistry, and chromatography technique (Xia et al. 2013; Akram et al. 2015; Shokoufi et al. 2020; Mao et al. 2021). However, these methods have shown some limitations as they require a qualified person to perform the analysis, are time-consuming, costly, and require different sample pre-treatments, restraining their potential use for quick, on-site, and real-time analytes detections. Table 3 shows the summary of the different traditional method that has been used for FQs detection with their advantages and disadvantages. When compared with various reported approaches, electrochemical methods, especially electrochemical biosensors have considerable benefits with their outstanding features such as less time consumption, cost-effectiveness, amenability to miniaturization, easy handling techniques, quick response, ability for on-site analysis, high sensitivity, and selectivity (Sun et al. 2023). Currently, significant attention has been drawn to electrochemical biosensor development for FQs detection. The next section will discuss more about the development of electrochemical biosensors.

**Table 3:** Summary of traditional technique used for FQs

Technique	Principle	Advantages	Disadvantages	Ref
Spectrophotometry	Here, light is passed through a sample and the fraction of light that passes through the sample is measured. The amount of light absorbed is called absorbance and the light passed through the sample is called transmittance	• Sensitive • Low-cost • Rapid analysis	• Low reproducibility • Less accurate • Limited applications	(Bonfilio et al. 2010; Kuntzleman and Jacobson 2016; Redasani et al. 2018)
Photochemistry	A photochemical reaction is triggered by the absorption of light and leads to an observable chemical change. After excitation by absorption of a photon of energy, a nuclear wave function is promoted from the electronic and nuclear ground state to an excited state. Thereby, creating a wave packet that evolves in time. This motion then directs the molecule toward a different geometry in the excited state	• Minimum undesirable decomposition • Low production of by-products	• Possible rapid termination of photoreactions • High unit capital input costs • High cost of photons and the light absorption	(Pape 1975)
Spectrofluorimety	Here, a fluorescence light is passed through a sample and the fraction of light that passes through the sample is measured. Fluorescence makes it more sensitive than spectrophotometry	• Highly sensitive • Quantification of some drugs in biological matrices	• Likelihood of analyzing only luminescent compounds • Complex analysis • Time-consuming • analysis • Failure of analysis of related substances	(Macii and Biver 2021)
Chromatography	In chromatography, the mobile phase (eluent) is passed through the column using gravity flow or a pump. The sample with several components is then introduced into one end of the column followed by elution with the mobile phase in the direction toward the other end. Different components will then move at different rates through the column depending on their partition or distribution coefficients between the mobile and the stationary phases. The separated components are collected at the other end of the column and detected	• Simplicity • Sensitivity • Accuracy • Reproducibility	• Requirement of high-cost equipment • Requirement for highly trained technicians • The need to be handled with care • Generation a lot of waste	(Mallik et al. 2016)

## Electrochemical biosensors for FQs

**Fig. 2:**
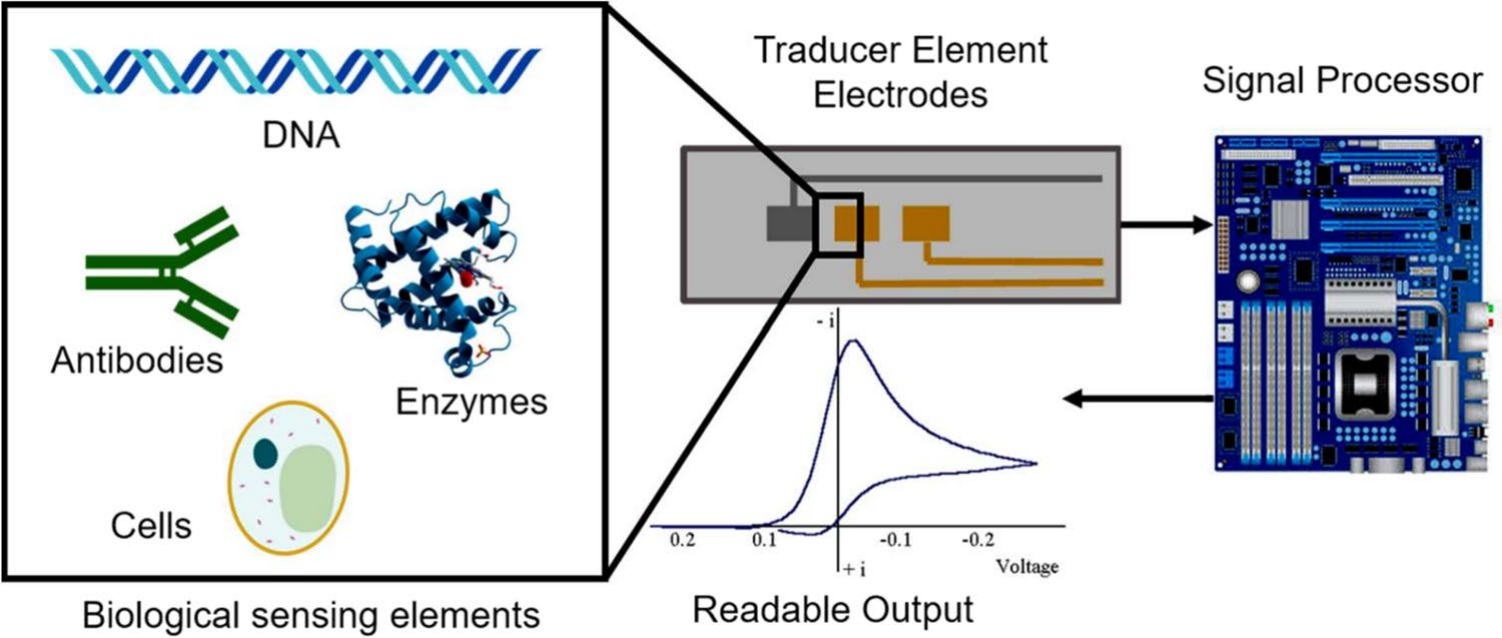
Schematic representation of an electrochemical biosensor (Hernandez-Vargas et al. 2018)

The need for the development and exploitation of analytical devices for the quantification, detection, and monitoring of antibiotics in water bodies has led to the development of sensing methods. The are various types of sensing techniques that have been successfully established for the detection of contaminants of emerging concern in food and water sample, including electrochemical sensors and biosensors, optical sensors, etc. (Kaur et al. 2020). Among these, electrochemical biosensors have currently attracted significant interest from various researchers as new promising sensing methods that can be employed in food analysis, health care, environmental monitoring, and drug delivery (Kumar and Neelam 2016; Shetti et al. 2019). They exhibit properties such as excellent sensitivity, rapid analysis time, and the possibility of miniaturization (Labib et al. 2016). The electrochemical biosensor comprises of biological recognition component (enzymes, DNA, antibodies and cells, etc.) that is directly connected to a chemical or physical transducer, which converts a chemical or biological signal into a measurable electrical signal (Akhavan et al. 2012; Dhar et al. 2019; Singh et al. 2021; Wang et al. 2023). In this type of biosensor, the electrochemical reaction takes place on the surface of the transducer between the bioreceptor and analyte producing detectable electrochemical signals with respect to voltage, current, impedance, and capacitance. The performance of electrochemical biosensor is influenced by a number of variables, including electrode type, electrolyte solution, and pH (Liu et al. 2019a). Among these, solution pH is one of the most important since, at various pH levels, not only the activity of an electrode is affected, but also the surface charges of the drug species vary, which has an impact on the behavior of the electrode surface during detection. (Wammer et al. 2013; Khosravikia and Rahbar-Kelishami 2022). Based on the electroanalytical technique that is used to measure chemical and biochemical interactions, biosensors are categorized as either potentiometric, impedimetric, amperometric or coulometric, conductometric and voltammetric (Hernandez-Vargas et al. 2018; Jalalian et al. 2018). Figure 2 shows the typical structure of biosensor comprising of three components, i.e., detector, traducer, and electronic component. The most critical characteristics of a biosensor development depend on its sensitivity and specificity. The specificity of biosensors is affected by the coupling efficiency between biological and transducer elements, whereas sensitivity is mainly influenced by the bioreceptor (Hernandez-Vargas et al. 2018). Recently, many researchers have based their focus on blending of nanostructured materials when development biosensor to enhance the sensitivity and detection limit. Numerous nanomaterials (NMs) and their composites such as gold nanoparticles (Au NPs), carbon nanotubes (CNTs), graphene (Gr) (Akhavan et al. 2014), and graphene–metal/metal oxide nanocomposites (Sethuraman et al. 2016) have been employed as perfect biosensor transducers, which offers great improvement in sensitivity by increasing electrochemical signal production and decreasing background noise (Shan et al. 2020). However, one of the most important factors for achieving an effective process during drug substrate detection is the electroosmotic flow through the nanopores/nanochannels within the NMs used for biosensor modification (Khosravikia 2023). In principle, the ion flow through the pore is changed when the analyte enters the pore under the applied potential, and this change is reflected over time in the current recording (Zhang et al. 2023). On other hand, there are certain challenges with the integration of NMs in electrochemical sensing because electrocatalyst materials (i.e., nanomaterials) slip off the electrode surface, affecting the performance of the biosensor (Baig et al. 2019; Liu and Mandler 2020). Although many researchers have solved these issues by employing nafion to stop these materials from falling off the electrode surface (Feleni et al. 2019, 2020; Wenninger et al. 2021), more study on this topic has to be done to enhance the surface kinetics of the electrodes. Review articles on electrochemical methods for the detection of antibiotics have been reported by a number of researchers (Tran et al. 2022; Ding et al. 2021; Seth and Rathinasabapathi 2022; Hong et al. 2023) and Table 4 shows the summary of biosensors used for FQs detections. This section discusses types of electrochemical biosensors that have been reported in literature.

**Table 4:** Summary of the biosensors used for FQs

Biosensor type	Analyte	Nanomaterials	Bioreceptors	Linear range (µM)	LOD (µM)	Ref
Optical	FQs	AuNRs	Antibody (Ab2)	0.13–29.91	0.31	(Dai et al. 2023)
Amperometric	Norfloxacin	CH-Y_2_O_3_	Antibody	1 × 10^−6^–10	3.87 × 10^−7^	(Yadav et al. 2020)
Electrochemical	Ciprofloxacin	nLa_2_O_3_ NPs	Antibody (Ab)	0.001–0.5	0.001	(Chaudhary et al. 2021)
Electrochemical	Ciprofloxacin	-	Aptamer	8.0 × 10^−4^–0.4	2.63 × 10^−4^	(Abnous et al. 2014)
Electrochemical	Ciprofloxacin	-	Aptamer	3 × 10^−4^–0.45	1.0 × 10^−5^	(Taghdisi Heidarian et al. 2021)
Fluorescent	Ciprofloxacin	-	Aptamer	0.01–0.2 10–120	0.051	(Jadhav et al. 2020)
Electrochemical	Ciprofloxacin	-	DNA	40–80	24	(Nawaz et al. 2006)
Electrochemical	Enrofloxacin	AuNPs/Ni-MOF	c-DNA	1 × 10^−2^–1 × 10^3^	5.6 × 10^−3^	(Lv et al. 2022)

### Enzymatic-based biosensor for FQs

Enzymes are biomolecules whose major constituents are amino acids and usually act as biocatalysts which are efficient to increase the biological reaction rate (Kurbanoglu et al. 2020). Biosensors that have enzyme molecules immobilized on transducing surfaces (electrodes) as biorecognition elements are referred to as enzymatic-based biosensors (Campaña et al. 2019). This type of biosensor operates on two main mechanisms, namely substrate detection and enzyme inhibition depending on the target analyte. The working principle of an enzyme-based biosensor relies on the catalytic reaction and binding abilities of the target analyte detection (Rocchitta et al. 2016). The are several possible mechanisms which are involved during the recognition process of an analyte. One of the mechanisms involves the metabolism of the analyte by the enzyme where the enzyme concentration is estimated by measuring the catalytic transformation of the analyte by the enzyme (Das et al. 2019). The other working principle is based on enzyme inhibition or activation by the target analyte to reduce enzyme activity. This process is based on the determination of enzyme activity in the presence and absence of inhibitor compounds (Asal et al. 2018). The decrease in product concentration provides the detection of inhibitory targets that inhibit the activity of certain enzymes. Enzyme-based biosensors can be developed on the basis of enzyme specificity (Cordeiro et al. 2018). These types of biosensors usually incorporate electrochemical, optical, and calorimetric transducers. Among them, electrochemical is the most used in literature. Enzyme-based biosensors have been widely used in different applications like the detection of industrial toxins and food contamination (Kurbanoglu et al. 2020), and viral, fungal, and bacterial disease detection (Kłos-Witkowska 2015). The most common family of enzymes with a well-recognized ability for the detection of different substances including pharmaceuticals are oxidases and peroxidases and have been widely used during enzyme-based biosensors development (Soylemez et al. 2019). The lifetime of an enzyme-based biosensor is dependent on the type of enzyme used as a recognition element.

### Photoelectrochemical (PEC) aptasensor for FQs

Biosensors that utilize aptamers as biorecognition component are known as aptasensors (Januarie et al. 2022). Aptamers are artificial functional single-stranded (ss) DNA or RNA molecules with the potential for precise recognition of specific and various targets, from small molecules to proteins and whole cells (Taghdisi Heidarian et al. 2021). Compared to antibodies, aptamers have several important characteristics such as low production cost, high stability in different physical and chemical form, long-term storage, easy modification, small size, and lack of immunogenicity and toxicity (Jin et al. 2019; Majdinasab et al. 2020). Fabrication of aptasensors is conventionally achieved through direct modification of a bio-functionalized sensor surface with aptamers using appropriate linkers or non-covalent modification of functionally activated surfaces with aptamers. NMs such as carbon fibers and Au NPs are mostly used during the development of aptasensors to enhance their sensitivity (He and Yan 2018; Li et al. 2018). In recent years, aptamer-based biosensors have emerged as a robust detection approach for antibiotic residues. For instance, Yang et al. (2021) developed a photoelectrochemical (PEC) aptasensor for Ciprofloxacin (CIP) detection based on Bi_24_O_31_C_l10_/BiOCl heterojunction. The PEC aptasensor achieved high sensitivity with a wide detection range (5.0 ~ 1.0 × 104 ng L^−1^) and low limit of detection (LOD) of (1.67 ng L^−1^, S/N = 3). Their method is straightforward and simple to follow. The PEC aptasensor was applied for the detection of CIP in water. Another photoelectrochemical aptasensor was developed by Zhang et al. (2021); they developed two materials with excellent PEC performance: three-dimensional nitrogen-doped graphene-loaded copper indium disulfide (CuInS_2_/3DNG) and Bi^3+−^doped black anatase titania nanoparticles decorated with reduced graphene oxide (Bi^3+/^B-TiO_2_/rGO). The aptasensor was based on applying different bias potentials to the two materials near one ITO electrode, the cathodic current generated by CuInS_2_/3DNH and the anodic current generated by Bi^3+/^B-TiO_2_/rGO was clearly distinguished without interfering with each other. Then, Enrofloxacin (ENR) and CIP aptamers were respectively modified onto the surface of CuInS_2_/3DNH and Bi^3+/^B-TiO_2_/rGO to construct a PEC aptasensor for sensitive detection of ENR and CIP. The aptasensor exhibit wide linear ranges of 0.01 to 10,000 ng mL^−1^ for ENR and 0.01 to 1000 ng mL^−1^ for CIP, with relatively low LOD 3.3 pg mL^−1^ for ENR and CIP in milk samples. In a study conducted by You et al. (2022), they developed a PEC aptasensor based on Ti_3_C_2_/Bi_4_VO_8_Br/TiO_2_ nanocomposite. The constructed PEC aptasensor presented an "on–off-on" detection signal and completed the specific detection of CIP in milk. With the increase of target detection concentration, the PEC aptasensor presented a detection range of 1 to 1500 nM and LOD of 0.3 nM. In Wu et al. (2022) study, they developed a self-powered microfluidic PEC aptasensor that uses photoactive AgBr/CuBi_2_O_4_ (ACO) composites as the photocathode matrix for ultrasensitive detection of CIP and ofloxacin (OFL). The ZnIn_2_S_4_-decorated CdS nanorod arrays (CZIS) as the photoanode was used instead of a platinum counter electrode to provide electrons. The CIP detection was accomplished through the steric hindrance effect in the photoanode due to the combination of aptamer_(CIP)_ and CIP. To increase the cathodic photocurrent intensity for OFL determination, a controlled release of luminol was first used. Luminol molecules were successfully embedded in the porous structure of silicon dioxide nanospheres (PSiO_2_) by the electrostatic adsorption between PSiO_2_ and aptamer _(OFL)_. The aptasensor exhibits wide linear ranges for CIP 0.001to 100 ng mL^−1^ and 0.0005 to 100 ng mL^−1^ for OFL detection. The LOD for CIP was 0.06 pg mL^−1^ and 0.022 pg mL^−1^ for OFL. Despite many advantages of aptamer, the interaction between aptamer and small molecules is more time-consuming and less specific compared to antibodies.

### Electrochemical immunosensor for FQs

**Fig. 3:**
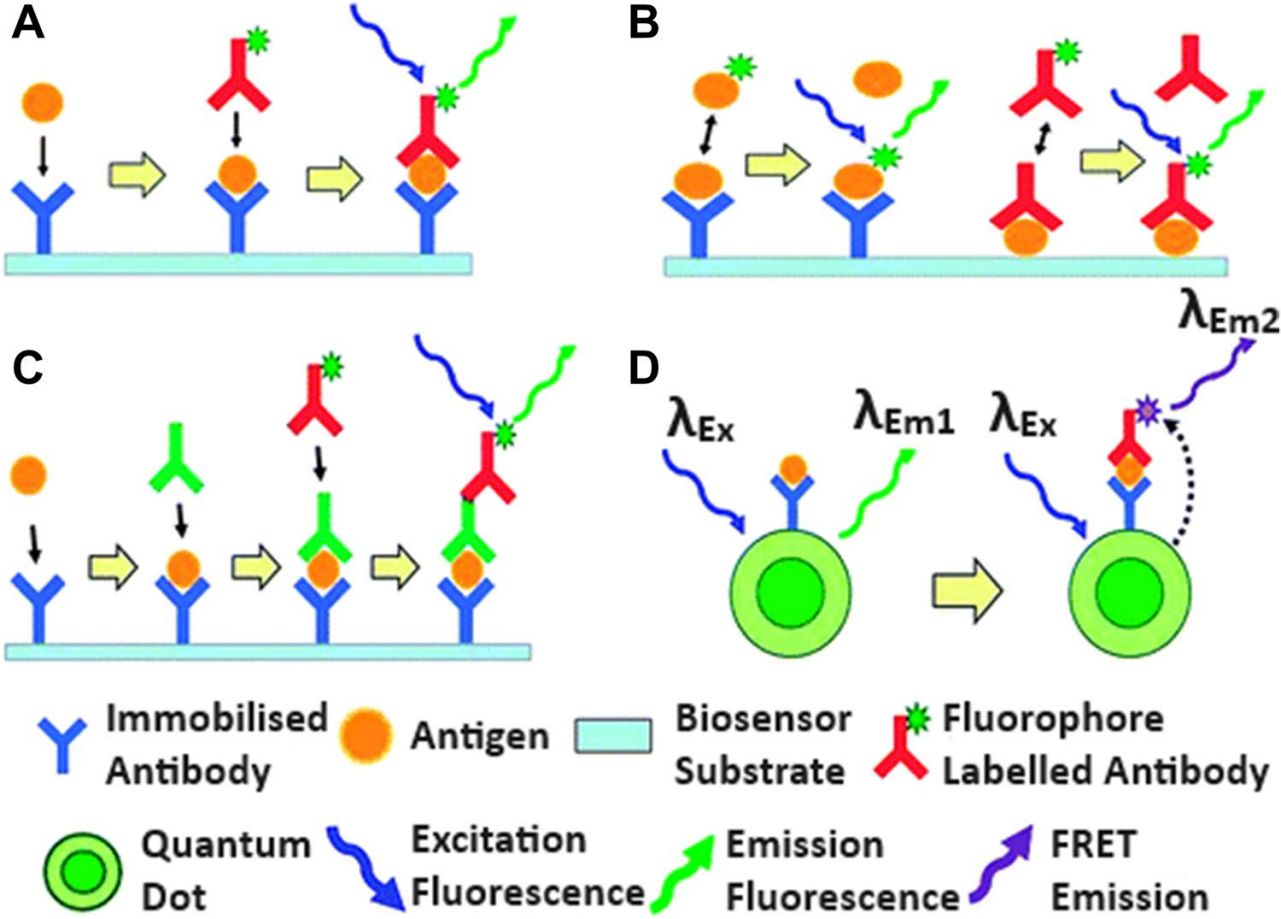
Possible immunoassay binding configurations are suitable for biosensing applications. Shown are the progressive reaction steps leading to the final binding structures for **A** sandwich structure formation using a fluorophore-labeled secondary antibody, **B** competitive style immunoassays using labeled antibodies/antigens, **C** extended sandwich structure formation using a fluorophore-labeled tertiary antibody, and **D** sandwich structure formation on a quantum dot (microparticle) surface using a secondary FRET-paired fluorophore (Mohammed and Desmulliez 2011)

Immunosensor is referred to as a biosensor that utilized an antibody as a biorecognition element. They have emerged as a powerful tool in clinical diagnostics, environmental monitoring, and food safety applications due to their extreme specificity. Immunosensors take advantage of high affinity of antibodies to antigens for the determination of specific analytes using an appropriate signal transducer. The working principle is reliant on detecting, processing, and displaying the signal produced by the formation of an antibody-antigen (Ab-Ag) complex. Figure 3 describes the possible mechanism for immunoassay binding configurations. Immunosensors play an important role in the detection of hazardous substances in foods. Aymard et al. (2022) constructed a dual electrochemical immunosensor for the detection of ENR in meat samples. Anti-quinolone antibody was immobilized onto screen-printed dual carbon electrodes via carbodiimide coupling. The detection principle was based on the competitive binding of this conjugate and free ENR on immobilized antibodies. The immunosensor was used to detect ENR at concentrations ranging from 0.005 µg. mL^−1^ to 0.01 µg. mL^−1^ and achieved the LOD of 0.003 µg. mL^−1^. The immunosensor was stable for at least 1 month at 4 °C and displayed a good specificity for other FQs drugs. Shinko et al. (2022) developed a piezoelectric immunosensor based on multi-walled carbon nanotubes (MWCNTs) for the detection of FQs in milk samples. They used MWCNTs in the formation of a stable piezoelectric sensor detection layer to increase the active specific surface area which is necessary for receptor molecule binding. The immunosensor achieved the LOD of 9 ng mL^−1^ and 8 ng mL^−1^ for levofloxacin (LEV) and CIP, respectively. Similarly, Bizina et al. (2022) developed a piezoelectric immunosensor with a recognition layer based on magnetic carbon nanocomposites for CIP detection in milk and meat sample. The receptor coating of the sensor was formed by the action of magnetic field on magnetic particles located on the surface of CNTs modified with a CIP conjugate. The immunosensor exhibits LOD of 2 ng mL^−1^ with a wide linear range from 5 to 400 ng mL^−1^. The use of magnetic carbon nanocomposites in the creation of a recognition layer ensured the reduction of a sensor preparation time. Lamarca et al. (2020) designed an impedimetric immunosensor to determine CIP in wastewater samples. They immobilized anti-CIP antibody on the surface of a printed carbon electrode. The observed Rct changes presented a linear relationship from CIP concentrations of 10^–5^ to 1.0 mg mL^−1^, with LOD and LOQ of 2.50 × 10^–6^ and 7.90 × 10^–6^ mg mL^−1^, respectively. The immunosensor presented high selectivity and repeatability, as well as a good recovery rate in wastewater samples (97%). Interference of the immunosensor with other compounds was not observed.

## Nanomaterial-based biosensors

Although enzymatic-based biosensor, aptasensor, and immunosensor are well established for the detection of various analytes, some challenges such as low stability, low sensitivity, and long detection time may still exist (Majdinasab et al. 2020). However, with the rapid development in nanotechnology, the application of NMs in sensing technology has become one of the most exciting forefront fields in analytical chemistry driven by their unique properties that offer excellent prospects for designing novel sensing systems and enhancing the performance of the bioanalytical assay (Ahmadipour et al. 2020; Chiam et al. 2022; Xia et al. 2012). Furthermore, NMs have the potential to increase the direct electron transfer (DET) and response time on a sensing system. For these reasons, nanomaterials-based biosensors have gained significant interest to many researchers owing to their distinctive properties such as high sensitivity, selectivity, low cost, and easy operation (Li et al. 2019). Since the performance of electrochemical biosensors largely depends on the performance of working electrodes (WEs), whereas the performance of WEs relies on the nature of the material used to modify the electrode (Wang et al. 2021), these NMs improve electron transfer among electrodes and detection species and behave as biocompatible frameworks for biomolecule control in electrochemical biosensors. A wide variety of NMs with different sizes, shapes, and compositions including carbon nanomaterials, metal nanoparticles, magnetic nanoparticles, quantum dots, and nanocomposites have been successfully used to develop electrochemical biosensors for antibiotics detection in food and water samples (Lan et al. 2017; Joshi and Kim 2020) and Table 5 shows the performance comparison of different nanomaterial-based electrochemical sensors for FQs. Herein, this section will mainly discuss nanomaterials for biosensor development.

**Table 5:** Performance comparison of different nanomaterial-based electrochemical sensors for FQs

Targeted analyte	Sensor	Electrode	Electrochemical technique	Real sample	Linear range (µM)	LOD (µM)	Recovery (%)	Ref
Norfloxacin	Ni/NiO/C/β-CD/RGO/GCE	^a^GCE	^e^CV	Water	0.4–80	0.01	-	(Cui et al. 2019)
Total FQs content	γ-CD-GQDs-CHI/SPCE	^b^SPCE	^f^DPV	Broths, bouillon cubes and milkshakes	4–250	1.2	90 to 106	(Bartolomé et al. 2023)
Norfloxacin	AuNPs-NOR-MIP/AuNPs/SPCE	SPCE	CV	Pharmaceuticals and aquaculture samples	0.00015 − 0.03113	0.15	-	(Vu et al. 2022)
Ciprofloxacin	N-prGO/CPE	^c^CPE	CV DPV	pharmaceutical	0.1–10	39		(Rahimpour et al. 2021)
Ofloxacin Norfloxacin	ERGO/GCE	GCE	CV DPV	Medicine and aquaculture wastewater	1–40 1–60	0.19 0.56	98.01 102.77	(Wang et al. 2022)
Enrofloxacin	TAPB-PDA-COFs/AuNPs/GCE	GCE	^g^SWV	Water samples and milk samples	0.05–10 10–120	0.041	96.7–102.2	(Lu et al. 2021b)
Ciprofloxacin	AuNPs/AC/GCE	GCE	CV DPV	Milk	0.5–25	0.20	78.6–110.2	(Gissawong et al. 2021)
Ofloxacin Pefloxacin Gatifloxacin	P-L-CuO: Tb^3+^ NS/GCE	GCE	DPV	Milk	0.01 and 800.0	1.9 2.3 1.2		(Taherizadeh et al. 2023)
Ciprofloxacin	Pt − RGO/GCE	GCE	DPV	Tap and river water	10–25	1.53		(Pham et al. 2022)
Ciprofloxacin	TiO_2_/PVA-GCE	GCE	DPV CV	Rainwater	10–120	0.04		(Zhao et al. 2021)
Pefloxacin	AuNPs/RGO/SWCNT/GCE	GCE	CV DPV	Milk	5.0 × 10^–7^–2.0 × 10^–5^	1.6 × 10^–8^		(Shi et al. 2020)

^*a*^*GCE*, glassy carbon electrode; ^*b*^*SPCEs*, screen-printed carbon electrode; ^*c*^*CPE*, carbon printed electrode; ^*d*^*AuE*, gold electrode; ^*e*^*CV*, cyclic voltammetry; ^*f*^*DPV*, differential pulse voltammetry; ^*g*^*SWV*, square wave voltammetry

### Carbon-based nanomaterials

Carbon-based nanomaterials (CNMs) offer various unique advantages when compared to many other NMs. They exhibit high surface-to-volume ratios, high electrical conductivity, long-term chemical stability, and enhanced mechanical strength. These characteristics enable CNMs to have lower detection limits and higher sensitivities (Qian et al. 2021). As a member of NMs, CNMs including carbon nanotubes (CNTs), graphene (Gr), graphene quantum dots (GQD), and carbon nanofibers (CNFs) have received great attention due to their exceptional physical, chemical, and electrical properties which has resulted in their widespread application during the development electrochemical biosensors for environmental monitoring, etc. (Power et al. 2018). Recently, most materials that are employed as electrocatalysts for the detection of FQs antibiotics are incorporated with CNMs to form advanced nanocomposites. Among them, CNTs and Gr are the most used CNMs in biosensors for antibiotics detection. Thus, the next section discusses the CNMs that are commonly used for electrochemical biosensors development.

#### Carbon nanotubes

Carbon nanotubes (CNTs) have been extensively used in various fields due to their unique electronic, optical, and catalytic properties (Ardani et al. 2022). The functionalization of CNTs with guest species either at their outer surfaces or in their nanochannels largely expands CNTs properties and applications (Jalal et al. 2019). Because CNTs have abundant surface area and surface characteristics, they can be physically or chemically altered to introduce a variety of materials, including biomacromolecules, active small molecules, and other materials, to their outer surfaces (Wang et al. 2012). As one-dimensional, seamless, and hollow graphitic NMs, CNTs are made of the sp2-hybridized carbon atoms bonding to each other through C–C σ interaction. CNTs were discovered by Iijima in 1991 (Iijima 1991). Since their discovery, CNTs have attracted considerable attention to the development of electrochemical sensors owing to their excellent conductivity and high tunability (Lawal 2016). CNTs offers the possibility of highly sensitive electrochemical sensors because of their electrical properties that enable the improvement of their analytical response (Cernat et al. 2015). Depending on the specific molecular structure and chemical composition of pharmaceutical compounds, CNTs may be used to promote the electron transfer of many reactions and facilitate the adsorption of organic molecules (Camilli and Passacantando 2018). Moreover, CNTs may produce synergetic effects that promote sensing (Jung et al. 2015; Mallakpour and Khadem 2016). To date, CNTs-based biosensors have been extensively used for the detection of FQs antibiotics. Liu et al. (2019a, b) designed an electrochemical sensor based on functionalized multi-walled carbon nanotubes (fMWCNTs) decorated with molecularly imprinted polymer (MIP) for the detection of norfloxacin (NOR). The porous structure of the fMWCNT 3D framework effectively increases the specific surface area, and the copolymer of fMWCNTs and MIP shows a synergistic effect for the electrocatalytic reaction of NOR on the modified sensor. The authors obtained wide linear ranges of 0.003 to 0.391 μM and 0.391 to 3.125 μM with LOD of 1.58 nM, and excellent selectivity in distinguishing NOR according to its structural analogs and possible interferences. The recoveries from pharmaceutical formulations ranging from 97.36 to 109.58% and the recoveries from rat plasma samples ranging from 83.00 to 115.67% were achieved. Another sensor for monitoring CIP was the fusion of nanocellulose and polypyrrole (NNC-PPY), incorporated with single-walled carbon nanotubes (SWCNTs) via a drop-casting method designed by Shalauddin et al. (2022) with a dynamic linear range of 1 to 50 µM and a LOD of 0.196 nM. Furthermore, the sensor exhibits a high sensitivity of 18.610 µA µM^−1^ cm^−2^, and the fabricated sensor was implemented successfully for the determination of CIP from water samples, biological fluids and pharmaceutical preparations. Sabeti et al. (2021) designed an electrochemical sensor based on a modified-glassy carbon electrode using f-MWCNTs and dopamine for the determination of CIP. The dopamine is electropolymerized to form a layer of polydopamine on the surface of functionalized CNTs. Two linear dynamic ranges from 0.075 to 10 µM and from 10 to 100 µM were obtained for CIP detection with LOD of 0.04 µM, and repeatability and reproducibility of 3.2 and 3.3%, respectively. Afterwards, the sensor’s selectivity against common interfering agents was checked out, and the sensor proved to be highly selective for CIP detection. The sensor was tested using urine samples for CIP detection. The sensor proved to be a trustable tool for CIP measurement in clinical and industrial applications.

#### Graphene

Graphene (Gr), is a two-dimensional (2D) CNMs, with a sheet of sp^2^ bonded carbon atoms that are arranged into a rigid honeycomb lattice, exhibiting the highest mechanical strength among the known materials, extraordinary electron transfer capabilities, excellent electrical conductivity, ultra-large specific surface area, unprecedented pliability and permeability, and favorable biocompatibility (Wang et al. 2016). Gr has become a novel and promising material for nanoelectronics due to its electrocatalytic activity, and it has been investigated as an electrode material for sensing devices. Gr has large surface area, good conductivity, and strong mechanical properties, but the major disadvantage lies in its poor dispersion. Pan et al. (2021) developed an electrochemical biosensor by the modification of a screen-printed carbon electrode (SPCEs) with graphene oxide (GO) for CIP detection based on the complexation of CIP with Mn^2+^ in the milk sample. The fabricated sensor achieved the linear range from 1.0 to 8.0 μM with LOD of 0.30 μM. The CIP recoveries in the milk samples ranged of 81.0 to 95.4% with relative standard deviations (RSDs) below 4.6%. Liu et al. (2023) designed an electrochemical sensor for the detection of OFL in water by depositing β-cyclodextrin (β-CD) and samarium oxide nanoparticles (Sm_2_O_3_ NPs) onto a laser-induced graphene (LIGr) electrode. The sensor obtains a wide linear range of 0.01 to 1.0 μmol L^−1^ and 1.0 to 120 μmol L^−1^ with low LOD of 0.005 μmol L^−1^ and good anti-interference ability and stability. This sensor was successfully applied in tap water for OFL detection.

### Metal nanoparticles

Besides the CNMs, metal and metal oxide nanoparticles have been widely used in electrochemical sensing materials for a long time due to beneficial features such as their small size; unique chemical, physical, and electronic properties, flexibility in fabricating novel and improved sensing devices, and good sensitivity to the ambient conditions and the ability to immobilize bioreceptors without affecting their bioactivity (Shrivastava et al. 2016). In addition, they offer exclusive physical, chemical, and electronic properties that make them suitable as transducer components of an electrochemical biosensor. Moreover, their surfaces are easy to functionalize. Metal nanoparticles are promising immobilization matrix for aptamers, proteins, antibodies, and enzymes (Joshi and Kim 2020). Among all metal nanoparticles, (Au NPs) have been widely explored to improve the LOD in electrochemical biosensing. Very often, AuNPs are associated with carbon materials such as CNTs and Gr in a synergetic effect to enhance the electrocatalytic effect of the working electrode (Rotariu et al. 2016). Thus, considerable effort has been devoted to metal nanoparticle–based electrochemical biosensors for antibiotics detection.

### Magnetic nanoparticles

In recent years, magnetic nanoparticles (MNPs) have received increasing attention toward the development of biosensor and their applications. The magnetic properties of MNPs are associated with the core and shell, which is active in biomolecule recognition, binding, and catalytic processes (Asab et al. 2020). MNPs display superparamagnetic properties at high temperatures. Superparamagnetic is when the net magnetic dipoles are zero (Mohammed et al. 2017). Most applications that use magnetic nanoparticles depend on the use of magnetic fields to manipulate their properties, which depends on the effectiveness of the particle magnetic moment and the field gradient. Moreover, MNPs can be integrated into the transducer materials, attracting analytes in the samples by an external magnetic field (Ventura-Aguilar et al. 2023). Compared to the non-MNPs-based biosensor, the biosensing strategy based on MNPs offers various advantages that include, improved sensitivity, lower detection limit, less noise, and quicker analysis (Calcaterra et al. 2022).

### Quantum dots

Quantum dots (QDs) are quasi-zero-dimensional semiconductor nanostructures that bind excitons in three spatial directions, and their quantum confinement effects result in good photoelectric properties. QDs are a type of novel fluorescent nanomaterial consisting of inorganic nuclei with organic molecules in the nanoscale range of 1–10 nm applied to the surface of the nucleus (Rajendiran et al. 2019; Li et al. 2020). These materials usually consist of carbon, silicon, cadmium selenide, cadmium sulfide, or indium arsenide and emit fluorescence when excited by a light source (Zhou et al. 2018). QDs possess unique chemical properties and excellent optical properties, including extended fluorescence lifetime, adjustable particle sizes, superior signal brightness, emission of multiple fluorescence colors, confined emission spectra, and broad excitation spectra (Ding et al. 2022). Currently, QDs have been recognized as an ideal material for the development of biosensors for antibiotic detection. According to the literature, QDs have been flourishing as promising tools in the development of biosensors for FQs detection.

### Nanocomposite

To overcome the limitations of individual NMs and homogenous preparations, a range of highly efficient approaches to synthesize various nanocomposites have been developed. Nanocomposite-based electrochemical biosensors have different applications in the field of environmental monitoring. Composite nanomaterials are composed of different functional components, and have garnered significant interest from materials scientists due to their combined physicochemical properties and great potential applications in the areas of electronics, photonics, catalysis, biotechnology, and nanotechnology (Hussain et al. 2017; Khasawneh et al. 2021). Generally, these nanocomposites exhibit a core/shell or a binary nanostructure which can be modified with different charges, reactive groups, or functional moieties on the surface with enhanced stability and compatibility (Pang et al. 2022). The successful application of such nanocomposites is highly dependent on their nanostructure, composition, stability, and dispersity of the particles under a range of different conditions. Furthermore, metal-based NMs can be combined with CNTs, Gr, rGO, polymers, etc., to develop nanocomposite material (Ahmadipour et al. 2021) and used to immobilize enzymes, antibodies, aptamers, etc. (Kucherenko et al. 2019). Therefore, many researchers have focused on the fabrication of different nanocomposite materials to develop novel multi-functional materials that possess serendipitous properties. Suanchan et al. (2021) developed a nanocomposite optosensing probe based on hierarchical porous carbon and graphene quantum dots incorporated with selective polymer for the detection of trace OFL. The probe showed a good linear range from 0.10 to 25 μg L^−1^ for OFL with LOD of 0.066 μg L^−1^. The probe was applied to detect OFL in milk achieving recoveries in the range of 92 to 99% with an RSD < 7%. In another study by Li et al. (2022), an ultrasensitive label-free molecularly imprinted polymer (MIP) voltammetric sensor for the selective determination of NOR, based on Au nanoparticle-functionalized black phosphorus nanosheet nanocomposite (BPNS-AuNP) covered by a polypyrrole-imprinted film was developed. The BPNS-AuNPs were found to improve the ambient stability and electrocatalytic activity, providing a large surface area for locating a higher number of specific recognition sites. The fabricated MIP/BPNS-AuNP/GCE sensor showed excellent sensing performance toward NOR, with a wide linear range from 0.1 nM to10 μM, with an extremely low LOD of 0.012 nM. Similarly, Jalal et al. (2019) also developed an electrochemical sensor based on a nanocomposite of MWCNTs, magnetite nanoparticle (Fe_3_O_4_), and polyethylenimine (PEI) for highly sensitive detection of CIP drug in biological samples and pharmaceutical formulations. Due to the high conductivity of CNTs and moderate conductivity of the polymer along with rich active sites of amine functional groups, the PEI@Fe_3_O_4_@CNTs nanocomposite displayed excellent electro-catalytic effect on the electro-oxidation of CIP. The electrochemical responses of the modified electrode were proportional to the drug concentrations in the range of 0.03–70.0 μmol L^−1^ with a limit of detection of 3.0 nmol L^−1^. Furthermore, the sensor was applied to determine CIP in the drug tablets, urine, and serum samples with acceptable recoveries of 97 to 108% and satisfactory precisions (1–3%RSD). Ghanbari et al. (2019) developed an electrochemical sensor for LEV detection based on poly (l-Cysteine) @AuNPs @ reduced graphene oxide nanocomposite. The sensor exhibits the linear response in two concentration windows of 1.0 × 10^−11^ M and 1.0 × 10^−4^, with LOD of 3.0 × 10^−12^ M. The glassy carbon electrode (GCE) modified through coating with a film of poly(l-cys)/AuNPs/rGO/GCE was found to offer high stability, reproducibility, and repeatability, as well as selectivity and was successfully used in the analysis of the LEV in synthetic blood serum, with recovery values of around 99%. In another study conducted by Bano et al. (2022), a novel PPy/Bi_2_MoO_6_/chitosan nanocomposites was prepared for electrochemical detection of CIP and benzene. The as-prepared sensor has shown a distinct increase in electrocatalytic and electrochemical activity. Furthermore, experimental results have confirmed high sensitivity due to the increased surface area and electron mobility of the electrocatalyst. The linear concentration falls into two distinct ranges for CIP with low LOD value and high sensitivity 0.01 to 1500 μM.

## General conclusions, challenges, and future perspective

FQs are important class of antibiotics that has received widespread in clinical application. The overuse of these drugs in medicinal treatment has become problematic due to the negative effects they pose to the environment. Thus, to reduce the excessive presence of FQs in water ways, research on the development of effective detection strategy is significant to keep the environment safe. However, due to the complexity of wastewater sample, the residual detection of FQs at trace level remains difficult. Therefore, the present review was compiled to report the recent advancements in FQs detection strategies in water samples. Compared with other reported technologies, electrochemical methods are relatively simple and portable. They are promising analytical tools for FQs detection owing to their admirable properties such as low cost, high sensitivity, selectivity, and specificity. On the other hand, NMs have been successfully utilized in developing ultrasensitive electrochemical biosensors for antibiotic detection as highlighted in this review. Although these electrochemical biosensing methods have some advantages over traditional methods, there are still some challenges associated with the detection of real water samples. Electrochemical biosensors are usually designed to detect one or two analytes at a time, while there are thousands of pollutants in the environment. In addition, the electrodes that are currently produced cannot simultaneously detect all the pollutants of interest. The presence of other pollutants can interfere with targeted antibiotics. Based on the above discussion, we can conclude that the electrochemical method for the detection of antibiotics is simple and cheap, but further improvement is needed in terms of anti-interference ability and sensitivity. Furthermore, we anticipate that developing electrochemical biosensors with NMs is a powerful tool in environmental monitoring, food safety, and medical diagnosis, as well as other fields. Lastly, we believe that nanomaterial-based electrochemical biosensors for FQs can offer higher sensitivity, higher speed and integration that will increase portability and automation. However, the issue of NMs being leached off the electrode is still of great concern and alternative measures need to be developed. Hence, future research on electrochemical detection methods for antibiotics could be based on these aspects.

## Data Availability

The datasets used and/or analyzed in this review are available on request from the corresponding authors.
